# Clinical outcome of narrow diameter dental implants: a 3-year retrospective study

**DOI:** 10.1186/s40902-023-00394-6

**Published:** 2023-08-01

**Authors:** Jae-Eun Kim, Youngjae Yoon, Ahran Pae, Yong-Dae Kwon

**Affiliations:** 1 Department of Dentistry, Graduate School, Kyung Hee University, Seoul, Republic of Korea; 2 Department of Oral and Maxillofacial Surgery, College of Dentistry, Kyung Hee University, Seoul, Republic of Korea; 3 Department of Prosthodontics, College of Dentistry, Kyung Hee University, Seoul, Republic of Korea

**Keywords:** Dental implants, Retrospective studies, Survival rate, Mechanical complications, Narrow diameter

## Abstract

**Background:**

This study aimed to analyze the clinical outcome and complications of narrow-diameter dental implants (NDIs) (diameter ≤3.5 mm).

**Methods:**

The 274 NDIs that met the selection criteria from 2013 to 2018 were included in the retrospective study, and the survival rates (SVR) were compared. Mechanical complications included screw loosening and fractures of the implant components, such as the implant fixture, abutment, and prosthesis. In addition, marginal bone loss (MBL) was measured immediately after surgery and 1 year after loading.

**Results:**

The 3-year cumulative SVR was 92.4%. Nineteen fixtures failed during the follow-up. The failure rate was significantly higher (OR=4.573, *p*<0.05) in smokers and was significantly higher in osteoporosis patients (OR=3.420, *p*<0.05). The vertical and horizontal values of MBL were 0.33±0.32 mm and 0.18±0.17 mm, respectively. Mechanical complications included screw loosening (5.5%) and porcelain fracture (2.2%), but no fractures of the fixture or components were observed. The choice of titanium and zirconium (TiZr) alloy implant was significantly more frequent in the posterior region. Bone graft was significantly more frequently done in the anterior region.

**Conclusions:**

According to the high SVR and stability of NDIs, the findings of the study suggest that NDIs may be a replacement for regular diameter dental implants (RDIs) and the use of TiZr alloy could extend the indication of NDIs. In the esthetic area, contour augmentation may be a reason for increasing the frequency of bone grafts.

## Background

With the aging population, the demand for implants has increased. As a result, the choice of implants was expanded according to each patient’s condition, such as the patient’s bone quantity, density, and location of implant placement [1, 2].

When the use of regular diameter dental implants (RDIs) (>3.5 mm) is challenging because of narrow alveolar bone width or insufficient bone mass narrow diameter dental implants (NDIs) (≤3.5 mm) can be alternatively selected [3]. NDIs are classified as follows: category 1, diameter <3.0 mm, mini-implants; category 2, 3.0≤ diameter <3.3 mm; and category 3, 3.3≤ diameter ≤3.5 mm [4].

Due to their reduced diameter, NDIs can be preferred in atrophic ridges to minimize patients’ surgical burden and postoperative complications and treatment costs and time can also be minimized [5].

In addition, previous studies have shown survival rates (SVR) similar to those of RDIs, with the fracture or failure of the implant itself being extremely rare and with stability comparable to that of RDIs [6–8].

However, compared with RDIs, the surface area between the alveolar bone and the implant surface and the mechanical strength are decreased because of the smaller size; therefore, the osseointegration between the implant and the bone can be weakened [9, 10]. This may eventually cause mechanical complications, resulting in implant failure [11]. In addition, in previous studies related to NDIs, the degree of influence on the failure of the implant was different depending on various variables such as the material and type of the implant and the state of bone density [12–14]. However, studies on the diversity related to NDIs are still insufficient. The stability of posterior NDIs continues to be discussed [15, 16].

Therefore, this study aimed to analyze the SVR of NDIs according to each variable and evaluate the clinical use and stability of NDIs according to the type and frequency of complications during the follow-up period. As a secondary outcome, clinical outcomes in the posterior area and marginal bone loss (MBL) were observed and evaluated.

## Methods

### Study model

This study collected clinical data of patients who received dental implants at the Department of Oral and Maxillofacial Surgery, Kyung Hee University Dental Hospital, from January 2013 to December 2018, based on the date of fixture placement. Data on patients with NDIs (diameter ≤3.5 mm) included follow-up data up to 2020. The data of patients with at least 1 year of follow-up from the time of the final prosthesis were analyzed. Despite the placement of NDIs, patients with insufficient data during the process of reading clinical charts and radiographs or those follow-up periods of less than 1 year were excluded from the study. This study was conducted as a retrospective study, and various clinical data, such as patient demographics, types and distinctions of implants, and related complications, were collected based on the patient’s medical records. This study was approved by the Institutional Review Board of Kyung Hee University Dental Hospital (KH-DT20014).

### Collection of clinical data

Understanding the SVR for the clinical evaluation of dental implants is important. In this study, the implant failure criterion included the loss of the implant fixture and the conditions contrary to the success criteria [17]. The success criteria for dental implants are as follows: clinical immobility of implants, no evidence of peri-implant radiolucency and bone loss (>1.5mm), and no persistent pain, paresthesia, discomfort, or infection.

**Fig. 1:**
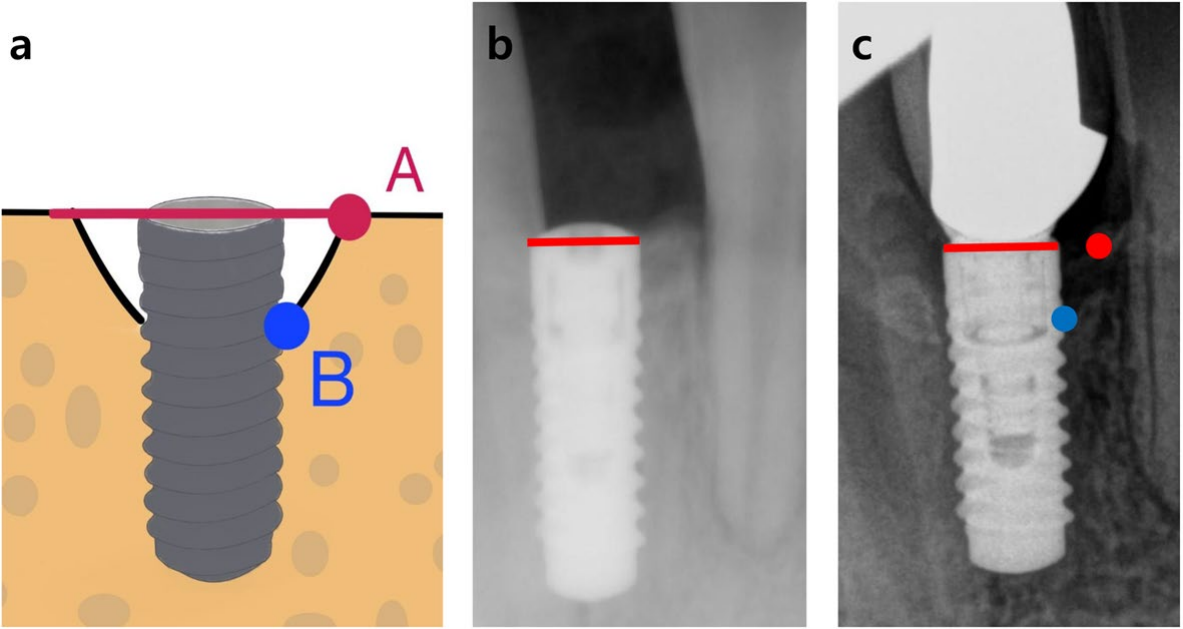
**a** Measurement of vertical and horizontal bone loss of peri-NDIs. **A** The point of first contact between the parallel extension line of the implant shoulder and the alveolar bone. **B** The contact point between the implant surface and the alveolar bone. **b** The postoperative clinical radiographs of NDIs. **c** The clinical radiographs of peri-implantitis

A radiographic examination was performed using periapical radiographs imaging with standardized digital radiographs during the follow-up period. The peri-implant bone level for NDIs was measured using software (ZeTTA PACS, TaeYoung Soft Co., Ltd, Anyang-si, Korea) at two-time points (immediately after implant fixture placement and 1 year after dental implant loading). It was applied using MBL measurement criteria as shown in Fig. 1 [18, 19]. MBL was measured twice a month by a participating researcher who was professionally trained in the field and recorded at four sites (mesial and distal vertical; mesial and distal horizontal). Cases with unclear radiographic images were excluded. Mechanical complications included screw loosening and fractures of the implant components, such as the implant fixture, abutment, and prosthesis.

### Statistical analysis

A demographic was summarized with mean and standard deviation values to understand the distribution of NDIs and their relationship with various variables. Kaplan–Meier survival curves with a log-rank test (95% confidence interval) were used to compare the cumulative SVR of NDIs. The Cox proportional hazard model statistical method was used to identify variables that affect implant SVR about certain systemic diseases. Peri-implant MBL for NDIs was compared using independent samples *t* test, Welch’s *t* test, and Kruskal-Wallis one-way analysis. The reliability of the MBL data was evaluated using the intraclass correlation coefficient [1, 2], more than 0.9 (*p*<0.05). Various comparisons according to the mechanical complications and variables of the NDIs placed in the posterior region were confirmed through statistical summaries and the chi-square test. Statistical tests were performed using the SPSS software (version 25.0; IBM Corp., Armonk, NY, USA). Statistical significance was set at *p*<0.05.

## Results

### Demographics

The data of 274 NDIs that met the study selection criteria from 2013 to 2018 are summarized in Tables 1 and 2. The patient’s major systemic diseases were summarized, and implant-related properties and mechanical complications were summarized with descriptive statistics. Among the selected patients, females accounted for the majority (65.3%), and the average age was 54 years old. The NDIs were placed relatively more in the anterior area (60.6%). The implant length varied from 8.0 to 13.0 mm, with 10.0 mm implants being the most used (78.5%). Implant diameters consisted of categories 2 and 3. Titanium-Zirconium (TiZr) alloy-type implants and chemically sandblasting with large grits and acid etching (mSLA) surface-treated implants were the most common, and screw loosening and porcelain fractures were observed as mechanical complications.

**Table 1:** Demographics of patients, *N* (%)

**Follow-up period**	
*M* (SD)/Min-Max	37.84(15.48)/12–76 months
**Sex**	
Male	95 (34.7)
Female	179 (65.3)
**Age**	
M (SD)/Min-Max	54.84 (18.50)/18–90
**Systemic condition**	
Hypertension	73 (26.6)
Diabetes mellitus	38 (13.9)
Osteoporosis	34 (12.4)
Smoking	43 (15.7)
Alcohol drinking	42 (15.3)

**Table 2:** Summary of NDIs, *N* (%)

Titanium type^*^	
TiZr alloy	148 (54.0)
Cp Ti	126 (46.0)
Surface treatment^*^	
SLA	69 (25.2)
mSLA	128 (46.7)
RBM	46 (16.8)
CA	31 (11.3)
**NDI category**	
Category 1 (<2.5mm)	0 (0.0)
Category 2 (2.5 to <3.3mm)	17 (6.2)
Category 3 (3.3 to 3.5mm)	257 (93.8)
**Length (mm)**	
<10	27 (9.9)
10	215 (78.5)
>10	32 (11.6)
**Abutment type**	
Stock	191 (69.7)
Custom	83 (30.3)
**Implant position**	
Incisor	119 (43.4)
Canine	47 (17.2)
Premolar	89 (32.5)
Molar	19 (6.9)
**Bone graft**	
Yes	122 (44.5)
No	152 (55.5)
**Mechanical complications**	
Screw loosening	15 (5.5)
Porcelain fracture	6 (2.2)

^*^TiZr alloy Titanium zirconium alloy, *Cp Ti* Commercially pure titanium

^*^SLA Sandblasting with large grits and acid etching, *mSLA* Chemically SLA, *RBM* Resorbable blast media, *CA* SLA with calcium chloride

### Survival and failure of NDIs

**Fig. 2:**
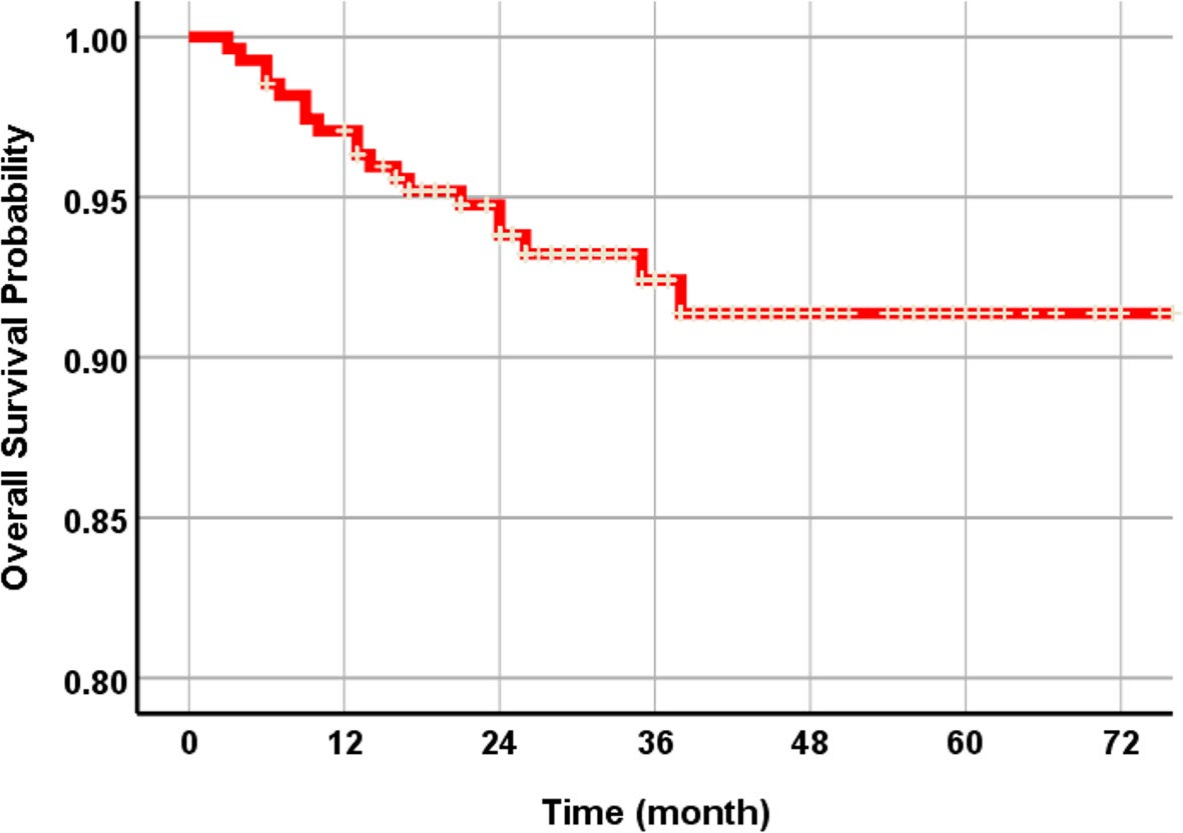
The Kaplan-Meier survival curves of all NDIs. The total 3-year cumulative SVR of 274 NDIs was 92.4%

**Fig. 3:**
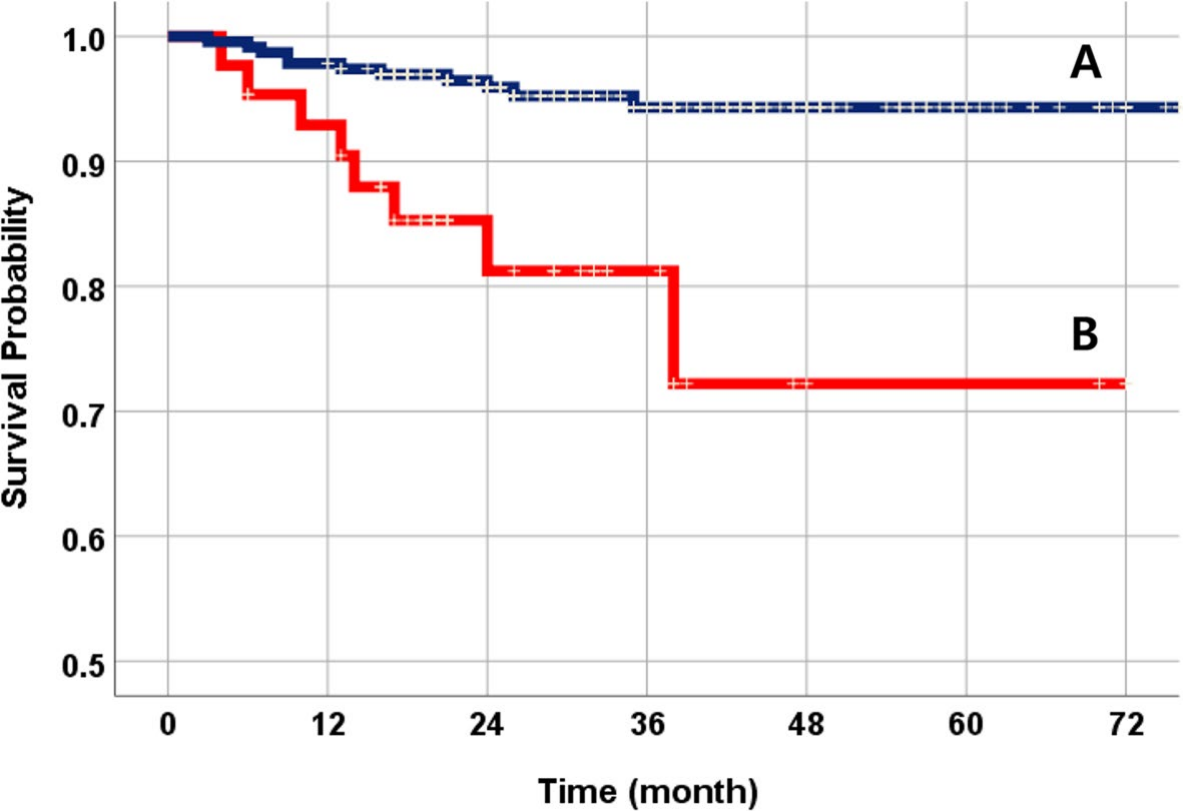
The NDI survival comparison curves by smoking status. **A** Non-smoker (95.2%). **B** Smoker (81.4%). There was a statistically significant difference (*p*<0.05)

**Fig. 4:**
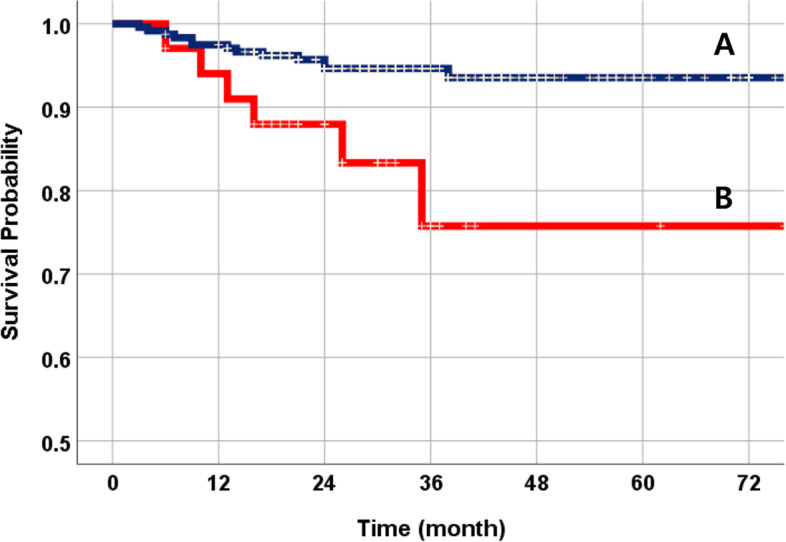
The NDI survival comparison curves by osteoporosis. **A** Non-osteoporosis (94.6%). **B** Osteoporosis (82.4%). There was a statistically significant difference (*p*<0.05)

**Fig. 5:**
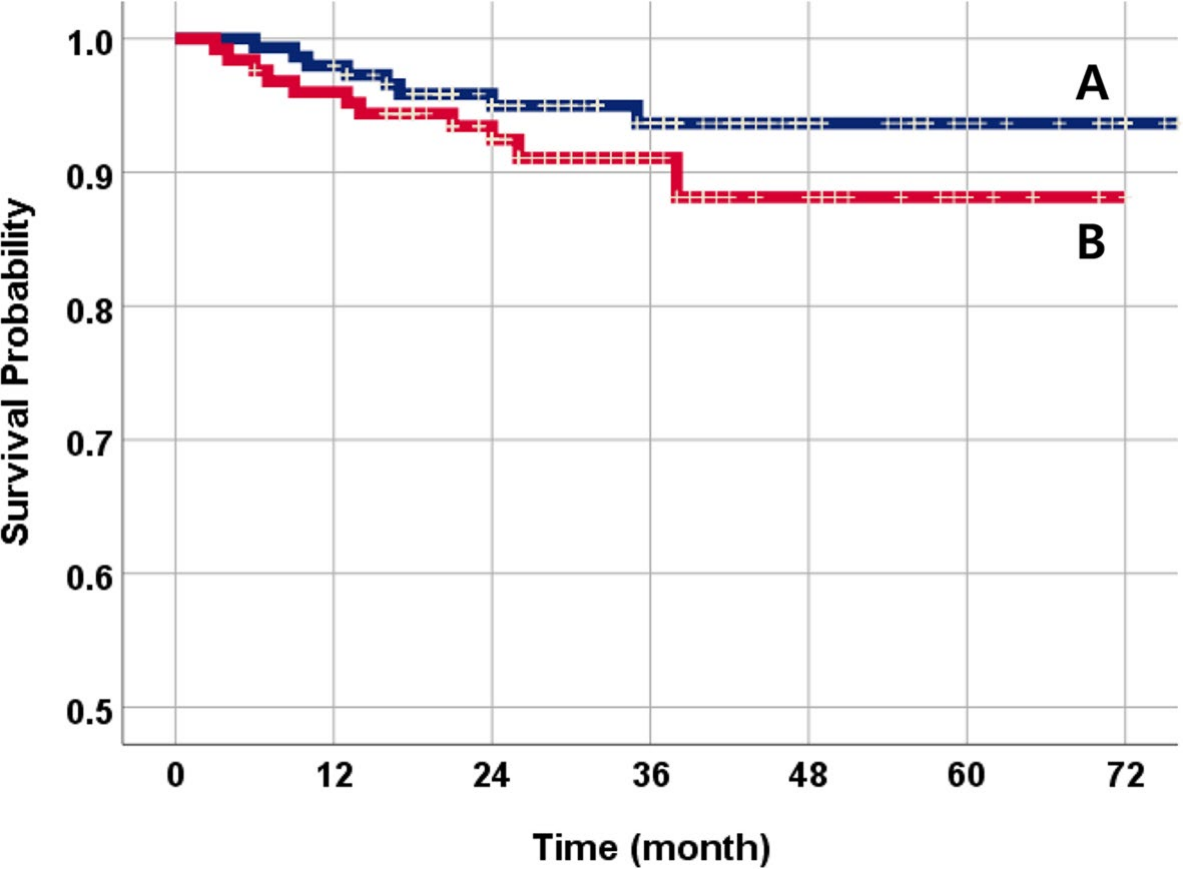
The NDI survival comparison curves by titanium type. **A** Ti-Zr alloy (94.6%). **B** cp Ti (91.3%)

The total 3-year cumulative SVR of 274 NDIs was 92.4% (Fig. 2). Nineteen fixtures failed during the follow-up period. Among them, 12 NDIs were initially dislodged due to failure of osseointegration. According to the implant success criteria, there were a total of 7 cases of peri-implantitis (Table 3). The SVR was compared using the following variables: systemic condition, titanium type, surface treatment, implant position, bone graft, and prosthetic splinted type. The SVR of NDIs in non-smokers was much higher than that of smokers (Fig. 3). Osteoporosis patients were more prone to implant failure (Fig. 4). As for the materials, the TiZr alloy showed high values of SVR (94.6%) (Fig. 5). Using the Cox proportional risk model statistical method, the influence of certain systemic diseases and implant characteristics on the SVR was evaluated. Smoking and osteoporosis were associated with implant SVR. Smokers showed a significantly higher failure rate (OR=4.758), and osteoporotic patients showed a significantly higher failure rate (OR=3.420) (*p*<0.05) (Table 4). There was no statistically significant difference between other systemic diseases and implant characteristics, type of prosthesis, and bone graft.

**Table 3:** Analysis of survival and failure of implants by year

Years	N^*^	Failure (*N*)	Cumulative SVR (%)	Detailed event (*N*)
0–1	274	8	97.0	8: Osseointegration failure
1–2	264	6	95.0	4: Osseointegration failure 2: Peri-implantitis
2–3	203	4	92.0	4: Peri-implantitis
3–4	104	1	91.0	1: Peri-implantitis
4–5	47	0	91.0	-
5–6	25	0	91.0	-

^*^N total number of NDIs—events and dropout data that occurred in the year

The Failure item represents the number of cases in which implant failure occurred out of the total number of *N*

**Table 4:** Cox proportional risk model table about implant survival

	SVR	OR	95% CI	*P value*
Osteoporosis				
No	94.6%	1 (reference)		
Yes	82.4%	3.420	1.286 - 9.098	0.014
Smoking				
No	95.2%	1 (reference)		
Yes	81.4%	4.573	1.820–11.489	0.001

This table represents only the variables that showed statistically significant results

### Marginal bone loss

**Fig. 6:**
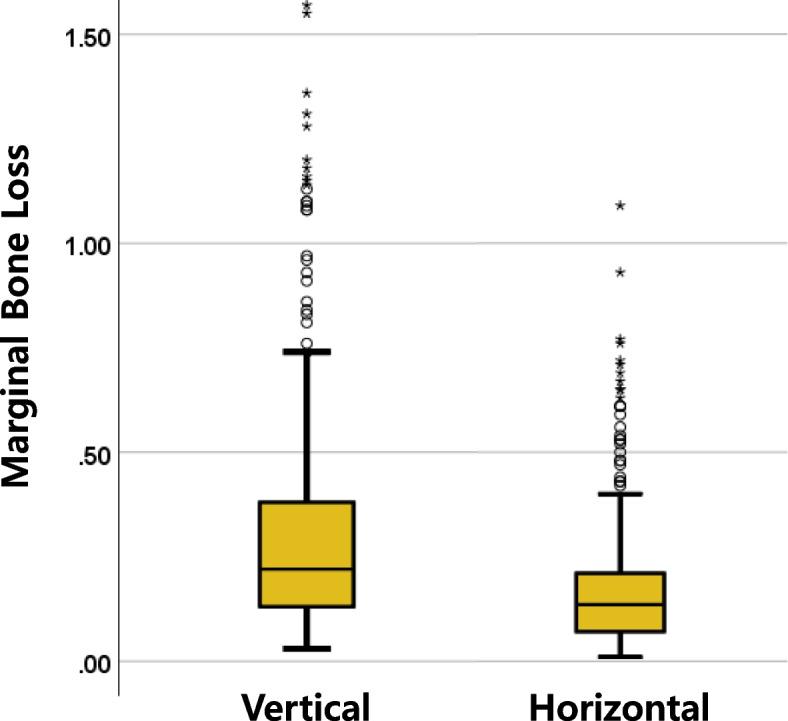
Overall vertical and horizontal MBL distribution of NDIs. MBL, immediately after implant fixture placement—1 year after dental implant loading. The mean values of vertical and horizontal MBL were 0.33±0.32 mm and 0.18±0.17 mm, respectively

The mean values of vertical and horizontal MBL were 0.33±0.32 mm and 0.18±0.17 mm, respectively (Fig. 6). Comparing the sub-items of each variable, the TiZr alloy type implant showed an average of 0.1 mm less bone loss, and the mSLA-treated implant showed less bone loss. Smokers showed the most bone loss, with an average of 0.72 mm and 0.38mm (Table 5).

**Table 5:** Comparison of MBL averages of NDIs according to conditions

	*N*	Vertical MBL(mm)		Horizontal MBL(mm)	
		M±SD	t/F(p)	M±SD	t/F(p)
NDIs	174	0.33±0.32	-	0.18±0.17	-
Implant					
Titanium type					
TiZr	148	0.29±0.26	–2.548 (0.012)^†^	0.16±0.16	–1.971 (0.050)
Cp Ti	126	0.38±0.36		0.21±0.19	
Surface treatment					
SLA	69	0.39±0.37^a^	12.696 (0.005)^†^	0.19±0.19	6.406 (0.093)^†^
mSLA	128	0.28±0.26^b^		0.16±0.16	
RBM	46	0.39±0.39^c^		0.20±0.17	
CA	31	0.35±0.22^d^		0.21±0.19	
Implant position					
Anterior	166	0.34±0.33	0.336 (0.737)	0.18±0.18	–0.126 (0.900)
Posterior	108	0.32±0.29		0.18±0.17	
Smoking					
Yes	43	0.72±0.35	8.253 (0.000)^†^	0.38±0.25	6.098 (0.000)^†^
No	231	0.26±0.25		0.15±0.13	

The mean comparison of MBL was performed using an independent sample *t* test

Kruskal-Wallis one-way test^†^, Welch’s *t* test^†^ (not equal variance, heteroscedasticity)

The post hoc analysis was conducted with the Dunn test, and there was a significant difference between groups b and c

### Mechanical complications

The mechanical complications included screw loosening (5.5%) and porcelain fracture (2.2%). There were no fractures of the implant fixture or abutment itself. There were three main characteristics of implants related to mechanical defects, which were more common in commercially pure titanium (CpTi) type implants and stock abutments. It was significantly more common in posterior implants, and screw loosening was the most common (Table 6).

**Table 6:** Frequency analysis of mechanical complications based on the materials, abutment type, and position

Mechanical complication						
	Titanium type		Implant position		Abutment type	
	TiZr alloy	Cp Ti	Anterior	Posterior	Stock	Custom
Yes	9 (42.9)	12 (57.1)	4 (19.0)	17 (81.0)	11 (52.4)	10 (47.6)
No	139 (54.9)	114 (45.1)	162 (64.0)	91 (36.0)	180 (71.1)	73 (28.9)
*χ*^2^/*p*	1.140^*^		0.000^***^		3.234^*^	

p^*^ < .1, p^***^ < .01

### Implant position

Mechanical complications were relatively common in the posterior region. In addition, TiZr alloy-type implants were selected more in the posterior region. Bone grafting was performed more frequently in the anterior region (Table 7). A total of 108 NDIs placed posteriorly, including the premolars and molars, were reported. The final prosthesis was a single or splinted fixed dental prosthesis (FDP). There were 38 splinted FDPs, 56 single FDPs, and 14 FDPs with pontics. Most of the NDIs were placed in the premolar position. The 3-year cumulative SVR of posterior NDIs was 91.7%. The vertical MBL of the posterior was 0.32±0.29 mm, and more bone loss was seen in the molar area than in the premolar area (Table 8).

**Table 7:** Mechanical complication, alloy type, and presence of bone graft according to implant position

	Anterior	Posterior	*χ*^2^, *p*
Mechanical complication^a^			
Yes	4 (2.4)	17 (15.7)	0.000^***^
No	161 (97.6)	91 (84.3)	
Titanium type			
TiZr alloy	81 (49.1)	67 (62.0)	4.407^**^
Cp Ti	84 (50.9)	41 (38.0)	
Bone graft			
Yes	86 (52.1)	35 (32.4)	10.280^***^
No	79 (47.9)	73 (67.6)	

p^**^ < .05, p^***^ < .01/Fisher’s exact test^a^ was performed (expected frequency less than 5 = 25%)

**Table 8:** SVR and MBL of the NDIs placed in the posterior region according to the restoration types

	Posterior	Premolar	Molar	*χ*^2^,* t*, *p*
NDIs (*N*)	108	89 (82.4)	19 (17.6)	
Splinted type in posterior				
Single type	56 (51.9)	43 (76.8)	13 (23.2)	4.246
Splinted type	38 (35.1)	32 (84.2)	6 (15.8)	
Splinted with pontics	14 (13.0)	14 (100.0)	0 (0.0)	
Marginal bone loss: M±SD(Max.-Min.)				
Vertical site	0.32±0.29 (0.03–1.69)	0.31±0.28	0.41±0.32	−1.368
Horizontal site	0.18±0.17 (0.01–0.76)	0.17±0.16	0.24±0.18	−1.694^*^
Survival rates(%)	91.7	92.1	89.5	0.036^**^

^*^The posterior region includes premolars and molars/p^*^ < .1, p^**^ < .05^*^

## Discussion

Herein, the SVR of NDIs according to each variable was compared, and the types of mechanical complications and stability in the posterior region were evaluated. The present study showed a high 3-year cumulative SVR of 274 NDIs, at 92.4%. In several previous studies related to NDIs, when the use of RDIs is challenging, or the need for additional surgery burdens patients from various perspectives, NDIs have been sufficiently proven to be suitable alternatives [20, 21]. Romeo et al. showed an SVR of 92%, with only a 5% difference compared with RDIs in the 7-year follow-up process [22], and Cordaro et al. and Degidi et al. demonstrated a high SVR rate of NDIs in long-term use, showing a 100% SVR after loading [23, 24]. Considering the results of this study, it suggests that NDIs may be used stably.

Herein, 12 implant fixtures were lost within the range of ≤1 year. This is regarded as a failure of osseointegration, and it is highly likely to have occurred because of decreased healing ability, anatomical condition, and premature overload [25]. The present study’s findings show that smokers and patients with osteoporosis have a 3 times higher NDI failure rate. Previous studies also reported that the rate of implant failure from smoking is 3 times higher and accounts for most implant failure factors [26, 27]. Osteoporosis may interfere with osseointegration during implant placement due to reduced bone density [28, 29].

In addition, some clinical studies have shown that hyperglycemia or insufficient blood supply may affect bone remodeling in patients with diabetes or cardiovascular disease [30]. This could also affect implant osseointegration. These systemic disease effects predict that the postoperative complications and healing period may increase when additional bone augmentation is performed. However, diabetes and hypertension did not appear to directly affect implant failure, and there was no correlation in the results of this study [31].

There have been studies comparing MBL at various time points. The marginal bone level increased due to bone remodeling for 1 year after implant placement [32], and an average of 1 mm of bone loss was reported to occur for 1 year after implant function [33].

According to one study, the largest change in MBL occurred for 1 year from the time of completion of the final prosthesis, and it was observed that it decreased gradually over the time [34]. In addition, MBL may be affected by some oral microbiomes [35]. This is a biologic process and many factors may be related to MBL like the patient’s oral habits, accurate match among implant components and 3-dimensional position of placement. Slightly lower values were observed in the posterior region in this study. Just as the SVR of implants in the maxilla and mandible with different bone densities can vary [36], it is necessary to consider and prepare for different effects depending on the implant position.

**Fig. 7:**
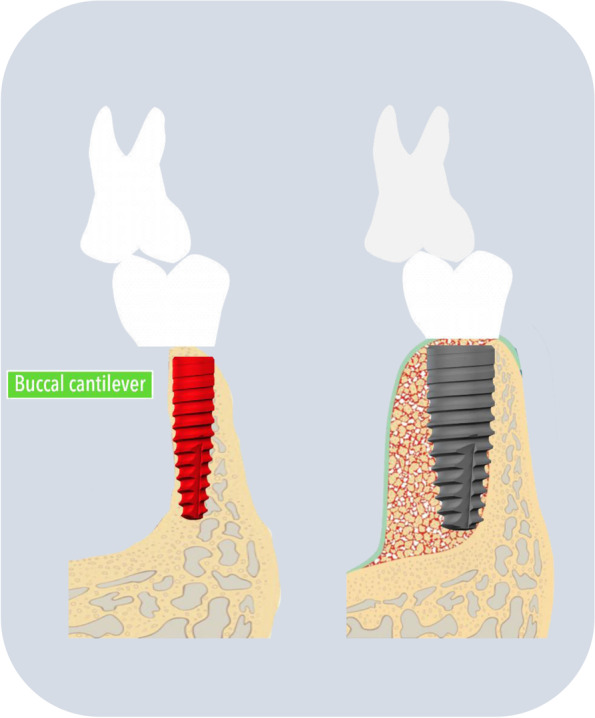
Association between NDIs and screw loosening. Due to the reduced diameter of NDIs, the force of masticatory is not evenly distributed. As a result, NDIs may be vulnerable to mechanical complications

The NDIs have structural limitations. Due to the reduced diameter of NDIs, NDIs can be more lingually placed than RDIs and the restoration can cause buccal cantilever and this may lead to mechanical complications like screw loosening (Fig. 7) although, in this study, less than 10% of mechanical complications occurred. As for the application of custom abutments, the use of custom abutments did not increase the frequency of mechanical complications and our result can be supported by other researchers and this may tell us modern CAD/CAM technology is accurate enough [37, 38].

The TiZr alloy-type implants showed relatively little bone loss and fewer mechanical complications. Also, TiZr alloy was more frequently selected in the posterior region compared to other groups. Because of the excellent mechanical strength of TiZr alloy, it is widely selected in the posterior region where the masticatory force is relatively strong. In addition to the results of previous studies that showed a higher SVR in TiZr alloy than pure titanium [39], it was found that bone augmentation can be avoided if NDIs can be applicable. Owing to the mechanical benefit of TiZr alloy, NDIs may be the first choice even in the posterior region. However, given that the TiZr NDIs in this study were preferentially placed in the premolar area, the routine use of an NDI for single molar replacement would be still premature and our result may corroborate the results of other researchers [40]; therefore, well-designed prospective studies should be performed.

Since NDIs are unavoidably selected in situations of narrow alveolar bone width or insufficient bone mass, they are often accompanied by bone grafting. Given that the utility of NDIs is to avoid bone grafts, it may be interesting to see that our data showed a higher frequency of bone grafts in the anterior region. To achieve esthetic gingival esthetics, a bone graft is usually recommended to reproduce the buccal contour. Since the depth of placement should be deep enough in the anterior region for aesthetic reasons and the surrounding soft tissue is abundant and thick, screw-retained restoration is usually preferred. The fixtures should be more lingually/palatally inclined. Therefore, a bone graft is often necessary to compensate for the labial side fenestration [41].

The safety of NDIs in posterior teeth with high masticatory load continues to be discussed. If RDIs are used along with bone grafting in areas where the amount of the bone is insufficient, the adhesion between the bone and the implant increases, which can have a positive effect on the maintenance of the implant from a physical point of view. However, as the number of patients with various systemic diseases increases, the postoperative complications and healing period become longer, which lowers the patient’s quality of life (QoL), making it difficult to use RDIs.

The posterior region showed a good MBL with a low bone grafting rate and a survival rate of over 90%, and there was no fracture of the fixture itself. Therefore, studies have shown that NDIs can function successfully without major biological and mechanical complications, and these functions may benefit elderly patients who need to avoid the burden of surgery.

Within the limitations of the retrospective study design, we could not include all interpretations of long-term follow-up patients due to the large number of dropouts. Therefore, it is judged that additional prospective studies are needed to evaluate the safety of post-mortem NDIs use. In addition, due to the limitations of non-randomized studies, it is necessary to consider and interpret the research environment in which bias cannot be controlled.

## Conclusion

In this retrospective study of NDIs, high SVR was observed. The MBL of NDIs showed a stable value, and there was no fracture of the implant structure itself. However, SVR was affected by systemic conditions such as osteoporosis and smoking, which resulted in the loss of NDIs due to the failure of initial implant fixation. These results may suggest that NDI can replace RDI and can be used stably if it is combined with patient-specific diagnosis considering the patient’s systematic condition.

## Data Availability

The datasets used and/or analyzed during the current study are available from the corresponding author on reasonable request.

## Abbreviations

RDIs: Regular diameter dental implants
NDIs: Narrow diameter dental implants
SVR: Survival rate
MBL: Marginal bone loss
TiZr: Titanium-Zirconium
CpTi: Commercially pure titanium
SLA: Sandblasting with large grits and acid etching
mSLA: Chemically sandblasting with large grits and acid etching
RBM: Resorbable blast media
CA: SLA with calcium chloride
FDP: Splinted fixed dental prosthesis
QoL: Quality of life

## References

[1] Odman J, Lekholm U, Jemt T, Thilander B (1994). Osseointegrated implants as orthodontic anchorage in the treatment of partially edentulous adult patients. Eur J Orthod.

[2] Andersen E, Saxegaard E, Knutsen BM, Haanaes HR (2001). A prospective clinical study evaluating the safety and effectiveness of narrow-diameter threaded implants in the anterior region of the maxilla. Int J Oral Maxillofac Implants.

[3] Schiegnitz E, Al-Nawas B (2018). Narrow-diameter implants: a systematic review and meta-analysis. Clin Oral Implants Res.

[4] Klein MO, Schiegnitz E, Al-Nawas B (2014). Systematic review on success of narrow-diameter dental implants. Int J Oral Maxillofac Implants.

[5] Schwarz MS. Mechanical complications of dental implants (2000) Clin Oral Implants Res 11 (Suppl 1):156-8. 10.1034/j.1600-0501.2000.011s1156.x 11168264

[6] Benic GI, Gallucci GO, Mokti M, Hammerle CH, Weber HP, Jung RE (2013). Titanium-zirconium narrow-diameter versus titanium regular-diameter implants for anterior and premolar single crowns: 1-year results of a randomized controlled clinical study. J Clin Periodontol.

[7] Zweers J, van Doornik A, Hogendorf EA, Quirynen M, Van der Weijden GA (2015). Clinical and radiographic evaluation of narrow- vs. regular-diameter dental implants: a 3-year follow-up. A retrospective study. Clin Oral Implants Res.

[8] Romeo E, Ghisolfi M, Rozza R, Chiapasco M, Lops D (2006). Short (8-mm) dental implants in the rehabilitation of partial and complete edentulism: a 3- to 14-year longitudinal study. Int J Prosthodont.

[9] Albrektsson T, Dahl E, Enbom L, Engevall S, Engquist B, Eriksson AR (1988). Osseointegrated oral implants. Journal of Periodontology.

[10] Adell R, Lekholm U, Grondahl K, Branemark PI, Lindstrom J, Jacobsson M (1990). Reconstruction of severely resorbed edentulous maxillae using osseointegrated fixtures in immediate autogenous bone grafts. Int J Oral Maxillofac Implants.

[11] Chrcanovic BR, Kisch J, Albrektsson T, Wennerberg A (2016). Factors influencing early dental implant failures. J Dent Res.

[12] Hallman M (2001). A prospective study of treatment of severely resorbed maxillae with narrow nonsubmerged implants: results after 1 year of loading. Int J Oral Maxillofac Implants.

[13] Jaffin RA, Berman CL (1991). The excessive loss of Branemark fixtures in type IV bone: a 5-year analysis. J Periodontol.

[14] Cochran DL (1999). A comparison of endosseous dental implant surfaces. Journal of Periodontology.

[15] Mohamed JB, Alam MN, Salman A, Chandrasekaran SC (2012). Narrow diameter implant in posterior region. J Indian Soc Periodontol.

[16] de Souza AB, Sukekava F, Tolentino L, Cesar-Neto JB, Garcez-Filho J, Araujo MG (2018). Narrow- and regular-diameter implants in the posterior region of the jaws to support single crowns: a 3-year split-mouth randomized clinical trial. Clin Oral Implants Res.

[17] Albrektsson T, Zarb G, Worthington P, Eriksson AR (1986). The long-term efficacy of currently used dental implants: a review and proposed criteria of success. Int J Oral Maxillofac Implants.

[18] Jung YC, Han CH, Lee KW (1996). A 1-year radiographic evaluation of marginal bone around dental implants. Int J Oral Maxillofac Implants.

[19] Duan X, Wu T, Xu X, Chen D, Mo A, Lei Y (2017). Smoking may lead to marginal bone loss around non-submerged implants during bone healing by altering salivary microbiome: a prospective study. J Periodontol.

[20] Alrabiah M (2019). Comparison of survival rate and crestal bone loss of narrow diameter dental implants versus regular dental implants: a systematic review and meta-analysis. J Investig Clin Dent.

[21] Ma M, Qi M, Zhang D, Liu H (2019). The clinical performance of narrow diameter implants versus regular diameter implants: a meta-analysis. J Oral Implantol.

[22] Romeo E, Lops D, Amorfini L, Chiapasco M, Ghisolfi M, Vogel G (2006). Clinical and radiographic evaluation of small-diameter (3.3-mm) implants followed for 1–7 years: a longitudinal study. Clin Oral Implants Res.

[23] Cordaro L, Torsello F, Mirisola Di Torresanto V, Rossini C (2006). Retrospective evaluation of mandibular incisor replacement with narrow neck implants. Clin Oral Implants Res.

[24] Degidi M, Piattelli A, Carinci F (2008). Clinical outcome of narrow diameter implants: a retrospective study of 510 implants. J Periodontol.

[25] Carlson B, Carlsson GE (1994). Prosthodontic complications in osseointegrated dental implant treatment. Int J Oral Maxillofac Implants.

[26] Bain CA, Moy PK (1993). The association between the failure of dental implants and cigarette smoking. Int J Oral Maxillofac Implants.

[27] Chrcanovic BR, Albrektsson T, Wennerberg A (2015). Smoking and dental implants: a systematic review and meta-analysis. J Dent.

[28] Starck WJ, Epker BN (1995). Failure of osseointegrated dental implants after diphosphonate therapy for osteoporosis: a case report. Int J Oral Maxillofac Implants.

[29] Giro G, Chambrone L, Goldstein A, Rodrigues JA, Zenobio E, Feres M (2015). Impact of osteoporosis in dental implants: a systematic review. World J Orthop.

[30] Donos N, Calciolari E (2014). Dental implants in patients affected by systemic diseases. Br Dent J.

[31] Anner R, Grossmann Y, Anner Y, Levin L (2010). Smoking, diabetes mellitus, periodontitis, and supportive periodontal treatment as factors associated with dental implant survival: a long-term retrospective evaluation of patients followed for up to 10 years. Implant Dent.

[32] Salamanca E, Lin JC, Tsai CY, Hsu YS, Huang HM, Teng NC (2017). Dental implant surrounding marginal bone level evaluation: platform switching versus platform matching-one-year retrospective study. Biomed Res Int.

[33] Adell R, Lekholm U, Rockler B, Branemark PI (1981). A 15-year study of osseointegrated implants in the treatment of the edentulous jaw. Int J Oral Surg.

[34] Arisan V, Bolukbasi N, Ersanli S, Ozdemir T (2010). Evaluation of 316 narrow diameter implants followed for 5–10 years: a clinical and radiographic retrospective study. Clin Oral Implants Res.

[35] Duan XB, Wu TX, Guo YC, Zhou XD, Lei YL, Xu X (2017). Marginal bone loss around non-submerged implants is associated with salivary microbiome during bone healing. Int J Oral Sci.

[36] Vigolo P, Givani A (2000). Clinical evaluation of single-tooth mini-implant restorations: a five-year retrospective study. J Prosthet Dent.

[37] Schepke U, Meijer HJ, Kerdijk W, Raghoebar GM, Cune M (2017). Stock versus CAD/CAM customized zirconia implant abutments - clinical and patient-based outcomes in a randomized controlled clinical trial. Clin Implant Dent Relat Res.

[38] Tartea DA, Ionescu M, Manolea HO, Mercut V, Obadan E, Amarascu MO (2023). Comparative study of dental custom CAD-CAM implant abutments and dental implant stock abutments. J Clin Med.

[39] Al-Nawas B, Bragger U, Meijer HJ, Naert I, Persson R, Perucchi A (2012). A double-blind randomized controlled trial (RCT) of titanium-13Zirconium versus titanium grade IV small-diameter bone level implants in edentulous mandibles–results from a 1-year observation period. Clin Implant Dent Relat Res.

[40] Becker J, Ferrari D, Mihatovic I, Sahm N, Schaer A, Schwarz F (2009). Stability of crestal bone level at platform-switched non-submerged titanium implants: a histomorphometrical study in dogs. J Clin Periodontol.

[41] Al-Nawas B, Domagala P, Fragola G, Freiberger P, Ortiz-Vigon A, Rousseau P (2015). A prospective noninterventional study to evaluate survival and success of reduced diameter implants made from titanium-zirconium alloy. J Oral Implantol.

